# Effects of game interactivity on perceived emotional challenge, mixed-affect emotional experiences and physiological responses

**DOI:** 10.3389/fpsyg.2026.1807290

**Published:** 2026-05-15

**Authors:** Tianyu Gao, Qi Song, Xurong Xie, Jian He, Xiaolan Peng, Jin Huang, Hui Chen, Chunxue Wang

**Affiliations:** 1 Human-computer Interaction Technology and Intelligent Information Processing Laboratory, Institute of Software, Chinese Academy of Sciences, Beijing, China; 2 University of Chinese Academy of Sciences, Beijing, China; 3 College of Computer Science and Technology, Beijing University of Technology, Beijing, China; 4 China National Clinical Research Center for Neurological Diseases, Beijing Tiantan Hospital, Capital Medical University, Beijing, China; 5 Department of Neuropsychiatry and Behavioral Neurology and Clinical Psychology, Capital Medical University, Beijing, China

**Keywords:** digital games, emotional challenge, interactivity, physiological signal analysis, player experience

## Abstract

**Introduction:**

As game genres have evolved, digital games have introduced a distinct type of user experience known as perceived emotional challenge, emerging from the integration of emotionally compelling narratives and interactive elements. While existing research has explored emotional challenge, the specific role of game interactivity in shaping this experience remains unclear, particularly regarding whether it amplifies subjective perceptions, mixed-affect emotional responses, and multimodal physiological reactions.

**Methods:**

A between-subjects experiment was conducted in which 28 participants with prior 3D game experience were allocated into two groups: a GamePlay group (*n* = 14) who actively played a carefully constructed scenario from *Fallout* 4 featuring emotionally compelling narratives, and a GameWatch group (*n* = 14) who passively watched a screen recording of the same scenario. Perceived emotional challenge (CORGIS), emotional responses (48-EARL), IDN-related user experiences, and immersion (IEQ) were assessed via self-report questionnaires. Peripheral physiological signals including electrocardiogram (ECG), electrodermal activity (EDA), respiratory activity (RSP), electromyography (EMG), and skin temperature (TEMP) were continuously recorded using Biopac MP150 sensors. Multimodal physiological features were extracted and subjected to both group-level t-tests and logistic regression with 10-fold cross-validation for condition discrimination.

**Results:**

Interactivity heightened perceived emotional challenge and significantly amplified specific hedonic emotional responses including contentment, joy, and pride, while reducing boredom and disappointment. GamePlay participants also reported stronger impression, role identification, emotional involvement, and challenge dimensions. At the physiological level, the GamePlay group exhibited higher heart rate, reduced beat-to-beat variability, shallower and less stable breathing, and stronger phasic electrodermal responses. A multimodal logistic regression model discriminated between the two conditions with 82.69% accuracy under 10-fold cross-validation, remaining robust after the removal of EMG (81.12%) and combined EMG and EDA features (76.06%).

**Discussion:**

These findings confirm that while emotionally compelling narratives alone can elicit perceived emotional challenge, game interactivity acts as a key amplifier, deepening hedonic and mixed-affect emotional experiences and producing a distinct and measurable physiological state. The multimodal physiological biomarkers identified in this study support the use of game-based paradigms for objective emotion assessment and provide empirical grounding for the development of adaptive systems aimed at personalized mental health monitoring and intervention.

## 1 Introduction

Games have great potential to evoke both entertaining and meaningful player experiences. Compared to other forms of media, games uniquely use the power of interactivity to allow users to engage with game content that otherwise might not have been possible (Iten et al., 2018). Foundational studies media psychology establish that interactivity is not merely a static attribute but a dynamic process that heavily mediates how users engage emotionally, cognitively, and physiologically with digital environments (Bogost, 2010; Kiousis, 2002; Oliver and Bartsch, 2010; Salen Tekinbas and Zimmerman, 2003). For instance, empirical evidence shows that active gameplay, compared to passive spectating, elicits stronger physiological arousal and emotional involvement (Juvrud et al., 2021; Kätsyri et al., 2013). Taken together, these findings suggest that gameplay can play a beneficial role in supporting emotion regulation and informing mental health monitoring (Granic et al., 2014; Villani et al., 2018; Kowal et al., 2021).

In recent years, digital games have introduced a new type of user experience—perceived emotional challenge—through the integration of emotionally compelling narratives and interactive elements. Unlike physical challenges (requiring speed or accuracy) or cognitive challenges (involving memory or reasoning) (Cox et al., 2012; Schell, 2008), emotional challenge requires players to overcome narrative tension or difficult themes through deep reflection and affective effort (Cole et al., 2015). Encounters with emotional challenges often put players in a more reflective state of mind, bridging the gap between momentary hedonic pleasure (e.g., fun and excitement) and deeper eudaimonic gratification (e.g., appreciation, self-reflection, and personal growth) (Bartsch and Hartmann, 2017; Daneels et al., 2021; Oliver and Bartsch, 2010). HCI research on emotional challenge has gained significant momentum, expanding to explore how affective game design patterns foster deeper player reflection (Cuerdo et al., 2024) and how emotionally challenging narratives can satisfy the core psychological needs of broader populations, such as older adults (Zhou et al., 2025).

To objectively capture these profound and often mixed-affect emotional states, researchers have increasingly turned to physiological signal analysis. In both passive media and interactive games, signals such as Electrodermal Activity (EDA), Electrocardiogram (ECG), and Heart Rate Variability (HRV) have proven effective in reflecting the intensity of emotional engagement, arousal, and cognitive load (Egger et al., 2019; Porter and Goolkasian, 2019; Soleymani et al., 2009). Recently, there has been a strong push toward leveraging multimodal physiological data to recognize game-experience responses across dynamic affective gaming environments (Ganiti-Roumeliotou et al., 2024), supported by the development of large-scale, open-access multimodal game corpora tailored for affective modeling (Barthet et al., 2024). These advancements offer new methodologies for automatically and objectively assessing players' emotional challenges (Peng et al., 2022).

Despite these advances, a critical gap remains: the specific role of interactivity in shaping emotional challenge is not well understood. Emotionally compelling narratives in modern games are fundamentally similar to the content delivered in films or television. Even without interactivity, these narratives can leave a deep impression and elicit profound emotions. Given the rapid growth of emotionally narrative-rich games, it is crucial to understand whether emotional challenge is primarily driven by the narrative content itself or significantly amplified by game interactivity.

To address this, we carefully selected a game scenario capable of inducing emotional challenge and controlled interactivity as the primary variable. We designed a between-subjects experiment comparing an active “GamePlay” group (playing a scenario in Fallout 4) with a passive “GameWatch” group (watching a screen recording of the same scenario). By measuring multifaceted self-reported player experiences alongside multimodal physiological responses, we aim to identify potential biomarkers associated with interactivity under emotionally compelling narratives.

Consequently, our three research questions are:

1. Does emotionally compelling narratives alone in digital games elicit perceived emotional challenge, or is it the incorporation of game interactivity that triggers this effect?
2. How does game interactivity affect players' mixed-affect emotional experiences in games featuring emotional challenge?
3. How do multimodal physiological responses differ between game watching and game playing, and are there objective physiological biomarkers that can distinguish these two conditions?

This paper clarifies the role of interactivity in digital games featuring emotional challenge. We demonstrate that active game playing provides a significantly stronger perception of emotional challenge than passive watching, fostering deeper eudaimonic emotional experiences. Furthermore, multi-modal physiological responses show significant differences between watching and playing, supporting the objective discrimination of these two conditions. These findings highlight the need for designers to create interactive mechanics that actively shape emotional challenges, and suggest that physiological signals can serve as objective indicators for distinguishing interactive from non-interactive gameplay conditions in ecologically valid settings.

## 2 Methods

The primary aim of the present study is to investigate the effects of game interactivity—specifically, the difference between playing games and watching others play—on perceived emotional challenge, mixed-affect emotional experiences, and physiological responses. By comparing these two modes of interaction, we aim to explore the distinct impact of interactivity on players in emotionally narrative-rich games, while also providing insights for future designs to deliver more unique and personalized gaming experiences.

In this study, we conducted a between-subjects experiment where we used game interactivity as an independent variable, with two groups of participants exposed to either a game-play (GamePlay) or a game-watch (GameWatch) experimental condition. In the group of GamePlay, participants directly engaged in playing a game scenario constructed carefully with three “Fallout 4” quests and influenced the game's progress and outcomes through their interactions. In the group of GameWatch, participants watch screen recordings of other players playing the same game scenario. In two groups, user experiences including perceived emotional challenge, multiple emotional responses, immersion, as well as experiences related to interactive digital narratives were assessed through self-report questionnaires. During the experiment, multiple physiological responses were recorded using a range of Biopac MP150 sensors that capture signals of electrocardiogram (ECG), electrodermal activity (EDA), respiratory activity (RSP), electromyography (EMG), and skin temperature (TEM) of each participant.

### 2.1 Materials

#### 2.1.1 Game scenario

Bethesda Softworks' “Fallout 4” was selected for our experiment because it is a popular first-person role-playing game with both rich narrative content and enjoyable playability. This open-world design and in-depth content provide a diverse environment, allowing us to obtain different game scenarios within a single game, catering to the needs of various research studies. “Fallout 4” has been widely used in previous studies and has been proven to be an effective choice for exploring player experiences related to emotional challenge (Peng et al., 2020; Cole and Gillies, 2022; Peng et al., 2022, 2023).

With “Fallout 4,” we carefully selected three main quests of Fallout 4 by two authors familiar with the game content and/or because of their expertise in the field of player experience and game challenge. The three main quests are: *Fire Support, Call to Arms* and *Blind Betrayal* game quests^1^. Then we constructed them as a coherent game scenario for our experiment. In particular, the construction spans a diverse array of game challenges and brings together emotionally compelling narratives from modern digital game genres, while also incorporating the rich interactivity and strategic depth characteristic of traditional game types. Table 1 illustrates the main events that players need to encounter within the game scenario. One of the authors who is pretty familiar with the game scenario segmented these main events.

**Table 1:** Main events of the game scenario.

Quest	Event	Description of events and instruction tips
	Tips	Read a brief instruction to learn the player's life experience of Vault 111.
		Learn that there is a big evil-force called Institution and they aim to create synths to control the destroyed world.
		Learn that there is another force of Brotherhood of Steel whose aim is to eliminate synths.
Fire support	1	Assist several soldiers to fight against a group of feral ghouls.
	2	Talk to the soldiers' commander, Paladin Danse, know that Danse work for Brotherhood of Steel and agrees toassist them to get a transmitter.
Call to arms	3	Follow Danse to walk.
	4	Fight against the robbers alongside with Danse.
	5	Follow Danse to walk, listen Danse to speak and may also fight against some wildlife on the way.
	6	Fight against the mad dogs alongside with Danse.
	7	Talk to Danse to learn the crime of synths in an abandoned rocket silo.
	8	Search for a password nearby to unlock a door in the way.
	9	Fight alongside with Danse against synths from one unlocked room.
	10	Fight alongside with Danse against synths from the other unlocked room.
	11	Fight alongside with Danse against the fire power of synths' weapon systems.
	12	Find the equipment to restore the power supply to the silo.
	13	Crack the password on the terminal to restore the power to the silo.
	14	Fight alongside with Danse against synths from the bottom of the silo.
	15	Talk to Danse and responding to join the Brotherhood.
Blind betrayal	16	Talk to Elder Maxson, be told Danse is a synth and be ordered to execute Danse.
	17	Talk to Proctor Quinlan and be confirmed that Danse is a synth.
	18	Talk to Scribe Haylen. Haylen says that Danse is a good friend. Be told where Danse is hiding.
	19-a	Talk to Danse until convincing Danse to escape.
	19-b	Talk to Danse until executing Danse.
	20-a	Talk to Maxson until persuading Maxson to spare Danse.
	20-b	Witness Maxson executing Danse.

Specifically, in the *Fire Support* quest, the player meets Paladin Danse, a commander of the Brotherhood of Steel in a battle against a swarm of feral ghouls. From Danse, the player learns that the Brotherhood is a just and united group and then responds to assist them to find a transmitter. In *Call to Arms*, the player follows Danse to an abandoned rocket silo to find the transmitter. Along the way, the player fights alongside Danse. In addition to encountering robbers and exploring the labyrinth-like silo, they go through many battles with the Brotherhood's biggest enemy—synths. Throughout the quest, Danse shows his resentment toward the synths. The *Blind Betrayal* quest takes place when the head of the Brotherhood, Elder Maxson, reveals to the player that Danse is a synth himself and orders the player to execute Danse. The player then confirms the news with Quinlan and locates Danse with Haylen. When the player finds Danse hiding in a bunker, the player then has a choice after talking to Danse about whether to save or execute him. If the player decides to save Danse, they need to convince Danse to escape with them. They then have to face Maxson when leaving the bunker. Again, the player must talk to Maxson to spare Danse, otherwise, Danse would be executed by Maxson.

#### 2.1.2 Settings of GamePlay and GameWatch

In the group of GamePlay, each participant wore headphones and used a keyboard and mouse to complete the three quest missions in sequence with a typical personal computer setting. In the playing, participants were allowed to use basic interactions and operations such as moving, shooting, searching, and interacting with non-player characters and key objects of the quests. Other advanced settings such as looting items, changing equipment and upgrading skills were not allowed.

In the group of GameWatch, each participant wore headphones but just watched the screen recording of others' playing with also the personal computer. The videos have a resolution of 1,920 × 1,080 and a frame rate of 36. As the game has two different endings, we prepared two different screen recordings: one with the result of executing Danse and the other with the result of sparing Danse. The proportion of population allocated with each kind of recording was Correspnding with the demographic of GamePlay group. In both groups, participants' multifaceted player experiences were measured and their peripheral physiological responses were also recorded with a Biopac MP150 device.

### 2.2 Measures

In this study, user experiences including perceived challenge, emotional responses, user experiences related to Interactive Digital Narratives (IDN), and Immersive Experience Questionnaire (IEQ) were measured with different metrics. The original English versions of all questionnaires were used, as all participants had sufficient English comprehension and expression ability to understand and complete them reliably. Additionally, peripheral physiological responses (ECG, EDA, RSP, EMG, and TEM) were continuously measured by using a range of Biopac MP150 sensors capturing.

#### 2.2.1 Perceived game challenge

The Challenge Originating from Recent Gameplay Interaction Scale (CORGIS) (Denisova et al., 2017, 2020), a 30-item questionnaire, was used in our study to measure four types of perceived challenge: cognitive, emotional, performative, and decision making challenge. Example items to measure perceived emotion challenge are “*This game is more than just a game to me”* and “*I felt a sense of responsibility for characters and events in the game*”. The CORGIS was especially developed and validated to comprehensively measure the experiences related to perceived challenge in modern digital games (Denisova et al., 2017, 2020). In our study, all items were scored on a 7-point Likert scale, where 0 refers to “Strongly disagree” and 6 to “Strongly agree.”

#### 2.2.2 Emotional responses

Players' various emotional responses were measured through the 48 EARL emotions rating method in which participants rate each emotional state on a 9-point Likert scale ranging from “Did not feel even the slightest bit” (0) to “The most you have felt in your life” (8). This method is modified with Gross's rating method of each kind of emotion (Gross and Levenson, 1995) and also combined with adoption of the Emotion Annotation and Representation Language (EARL) (Shaver et al., 1987). The rating method has been successfully used recently in assessing the wide range of emotional responses induced by emotionally challenging games (Peng et al., 2020) and serious data stories (Lan et al., 2022).

#### 2.2.3 IDN-related user experiences

As the experimental materials adopted in this study has quite similar characteristics to those of interactive digital narratives (IDN), we also adopted the IDN toolbox (Roth and Koenitz, 2016) (7-point Likert scale, 0 = strongly disagree and 6 = strongly agree) to evaluate several important dimensions of user experiences that our experimental materials may also evoke, such as the eudaimonic entertainment experience of appreciation (Oliver and Bartsch, 2010), role-identification (Oatley, 1995), and aesthetic-pleasantness (Wanzer et al., 2020). The IDN toolbox (Roth and Koenitz, 2016) was proposed in 2016 to specifically evaluate the multifaceted IDN user experiences related to interactive storytelling (Roth, 2016), psychology entertainment (Bryant and Vorderer, 2013) and Murray's theory framework (Page, 1999).

#### 2.2.4 Immersive experience questionnaire

To measure the immersion experienced by players or viewers during the experiment, the 31-item Immersive Experience Questionnaire (IEQ, 7-point Likert scale, 0 = not at all and 6 = a lot) (Jennett et al., 2008) was used, evaluating from five aspects: Cognitive involvement, Real-world dissociation, Control, Emotional involvement, and Challenge.

#### 2.2.5 Measures of physiological signals

**Figure 1:**
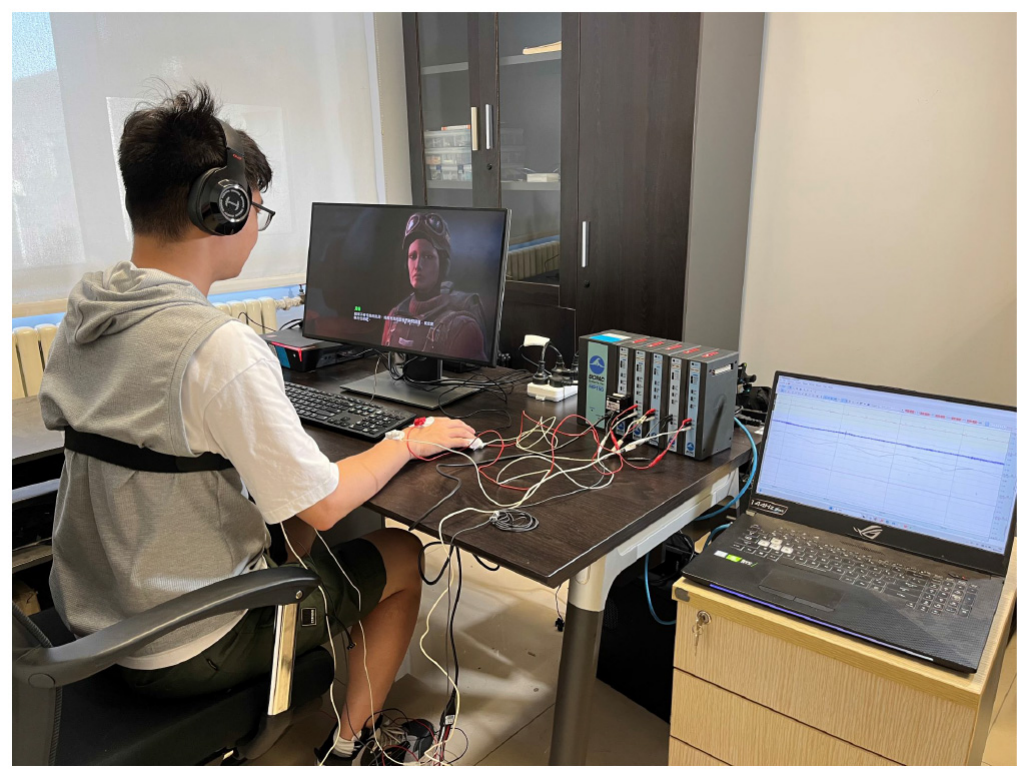
Experimental setting.

During the experiment, physiological signals include ECG, EDA, RSP, EMG, and TEM were recorded continuously using Biopac's MP150 at a sampling rate of 1000 Hz. ECG, EDA, and EMG are all measured using disposable AgCl electrodes. Skin temperature is collected using Biopac's TSD202B, and RSP data is collected using the BioNomadix respiratory sensor belt from Biopac. Figure 1 shows the experimental setting.

### 2.3 Participants

Regarding the difficulty for novices to play the game, twenty-eight participants with prior 3D gameplay experience but no previous exposure to the Fallout series were recruited for the experiment. To be included in the study, participants were required to be between 18 and 35 years old and to have some interest in video games. Participants were excluded if they reported visual, auditory, mental, or physical disorders, or a history of motion sickness. Because both psychological questionnaires and physiological measures were collected in this study, participants were also screened for self-reported mental and physical health conditions during recruitment. Participants were randomly assigned to the two independent groups. Educational background was broadly matched across groups, as all participants were university students. Finally, fourteen participants (12 males, two females, mean age = 24.07, standard deviation = 2.53) joined the GamePlay group, and another 14 participants (12 males, two females, mean age = 24.57, standard deviation = 2.53) joined the GameWatch group. This sample size aligns with methodological conventions in related research on physiological signals in game-based interactive environment (Peng et al., 2020, 2022, 2023; Guertin-Lahoud et al., 2023; Fijačko et al., 2025; Orozco-Mora et al., 2022; Niu et al., 2025), ensuring reliable detection of hypotheses. Results of Mann–Whitney *U*-Test showed that there was no difference between the two groups in terms of age (*Z* = −0.92, *p* = 0.36) and digital gameplay experience (*Z* = −0.07, *p* = 0.95).

### 2.4 Procedure

For each participant in the GamePlay group, written consent was obtained prior to a brief introduction on game operations and scenario backgrounds. Participants were then fitted with physiological sensors and underwent a short familiarization period with the controls. Subsequently, they played the complete game scenario (approximately 50 min), with their gameplay recorded and an experimenter nearby for assistance. Afterward, participants removed the sensors, completed multiple survey scales, and participated in a short interview. The entire experiment lasted roughly 90 min.

The GameWatch group followed the same procedure, except participants only watched a screen recording of the gameplay without interaction. In the GamePlay group, 12 participants chose to spare Danse, while 2 chose to execute him, leading to distinct narrative outcomes. Because this highly unequal distribution prevented meaningful statistical comparisons between the two decision paths, our analysis evaluates the emotional challenge of the quest as a unified whole. To ensure consistency, the GameWatch group watched recordings reflecting these two endings in the exact same 12:2 proportion.

### 2.5 Physiological data preparation

#### 2.5.1 Emotional challenge events selection and segmentation

Emotional challenge emerges after players have developed a sufficient level of narrative understanding and immersion, usually at critical moments in the game that involve ambiguous interpretation or require meaningful decision-making. In this study, with the game scenario we constructed, players need to encounter all the main game events listed in Table 1. Players first complete the game quests *Fire Support* and *Call to Arms*, where they gradually understand and engage with the narrative through scene exploration, interacting with NPCs, and event comprehension. Then, they proceed to the quest of *Blind Betrayal*, where they experience more emotionally charged narratives and critical moments that require meaningful choices—such as killing or sparing Danse—to evoke the experience of emotional challenge.

To precisely analyze physiological responses tied to emotional challenge experiences, we extracted and analyzed physiological signals exclusively during game events associated with emotional challenge. In this study, game events in the quest of *Blind Betrayal* (see Table 1) were identified as events tied to emotional challenge by two co-authors with expertise in game studies and emotional challenge. The two co-authors independently reviewed the narrative sequence of the quest and identified key events that involved emotionally difficult decisions or morally conflicted situations. Subsequently, the physiological signals tied to these events were carefully extracted and analyzed.

**Figure 2:**
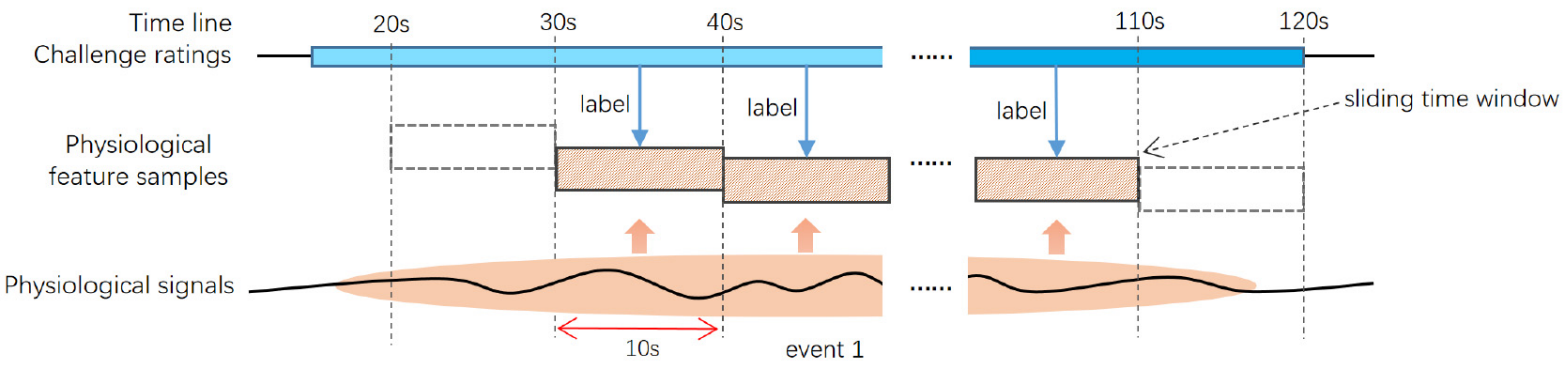
Physiological signal feature extraction.

Since each player's game progression differed, we reviewed their screen recordings to mark the start and end times of game events tied to emotional challenge. We then calculated each event's duration and identified the corresponding physiological signals associated with emotional challenge. For the GameWatch group, we similarly identified physiological signals that corresponded to the emotional challenge-related events in the game videos they observed. Finally, to extract physiological signals associated with emotional challenge and also eliminate any possible periods of physiological noise during the game, only feature frames located within the game event period tied to emotional challenge (marked by the start and end times) were deployed for detection (as shown in Figure 2).

#### 2.5.2 Physiological signal pre-processing

The preprocessing of physiological signals was conducted using Biopac^2^ AcqKnowledge software. For electrocardiogram (ECG) data, *z*-score normalization was first applied to reduce individual differences, followed by the use of a median filter with widths of 200 and 600 ms to remove baseline drift caused by participant movement or respiration (Hsu et al., 2017). Subsequently, the Pan-Tompkins QRS detector was used to identify R-wave peaks (Pan and Tompkins, 1985), and the time between consecutive R-wave peaks was calculated as RR intervals, serving as a measure of heart rate variability (HRV). For electrodermal activity (EDA) data, a high-pass filter with a cutoff frequency of 0.02 Hz was applied to eliminate baseline drift (Shukla et al., 2019). For respiratory signal (RSP) data, a band-pass filter with a range of 0.1–5 Hz was used to remove abnormal respiratory cycles. The Penh analysis in AcqKnowledge was utilized to detect respiratory cycles, with a threshold set at 65% of the exhaled volume.

#### 2.5.3 Feature extraction

Physiological signal features were extracted using Matlab for 80 different characteristics (see Appendix A). For each type of signal from every participant, z-score normalization was first applied to reduce individual differences. Then, physiological features for each frame were calculated using signals within a sliding time window of 10 s width and 10 s offset (see Figure 2). Although there is no consensus on the optimal size of the time window for peripheral physiological analysis (Yang et al., 2018), the most commonly used time windows are 60, 30, and 10 s (Kreibig, 2010). In this study, to delve deeper into the fine-grained dynamic physiological responses, the time window was set to 10 s wide. Additionally, to avoid statistical dependence between overlapping segments, a sliding offset of 10 s was used.

- The ECG signal underwent feature extraction by initially identifying R-wave peaks and calculating the RR intervals along with the first-order differences (Diffs) between consecutive tokens for time-domain analysis. The frequency-domain analysis involved resampling the RR intervals to 8 Hz using cubic spline interpolation (Niskanen et al., 2004), followed by applying the Fast Fourier Transform (FFT) to a 32-s window width frame containing 256 samples. The resulting frequency spectrum was divided into three bands: Very Low Frequency (VLF) from 0 to 0.04 Hz, Low Frequency (LF) from 0.04 to 0.15 Hz, and High Frequency (HF) from 0.15 to 0.4 Hz (Hsu et al., 2017). The power and Power Spectral Density (PSD) within these bands were extracted as features indicative of cardiac activity.
- The RSP data were processed similarly to the ECG for time-domain features, capturing the rhythmic patterns of breathing without additional frequency-domain analysis, given the focus on the temporal aspects of respiratory activity.
- For the TEM data, feature extraction focused on the amplitude of the temperature fluctuations and their first-order differences (Diffs), providing insights into the thermal responses of the body.
- The EMG signal was first down-sampled to 32 Hz to reduce the data rate while preserving the essential information. Subsequently, a 6-level wavelet decomposition using the Daubechies5 wavelet was applied to decompose the signal and extract features indicative of muscle activity (Cheng and Liu, 2008).
- The EDA signal was analyzed in both the time and frequency domains (Shukla et al., 2019). It was segmented into three frequency bands: Low Frequency (LF) from 0.02 to 0.5 Hz, High Frequency (HF) from 0.5 to 1 Hz, and Very High Frequency (VHF) for frequencies above 1 Hz.

### 2.6 Statistical analysis

For all data collected using self-reported questionnaires, the internal consistency reliability of each factor was assessed using Cronbach's α. As all the collected data meet the criteria for normal distribution, t-test was used to statistically assess the differences of the two experimental groups using SPSS. In all figures presented in this paper, * refers to *p* < 0.05, which indicates a significant difference between the two groups. In addition to the *p*-values, we report Cohen's ***d*** as a measure of effect size and the 95% Confidence Intervals (CI) of the difference to indicate the precision of the estimation.

For physiological analyses, features were first extracted from consecutive non-overlapping 10-s windows within events associated with emotinal challenges. This event-based analyasis of emotional challenge has also been adopted in previous study (Peng et al., 2019). For group-level comparisons, the window-level feature values were averaged within each event, yielding one event-level estimate per participant for each feature, and independent-samples *t*-tests were then conducted between the two groups.

To evaluate whether physiological responses could discriminate between the GamePlay and GameWatch conditions, logistic regression classifiers were trained on the full set of window-level physiological feature vectors. Model performance was evaluated using a 10-fold cross-validation procedure. This was performed in a participant-wise manner—ensuring that all data windows from a single participant were strictly assigned to the same fold—to prevent optimistic performance estimates caused by within-subject information leakage. Robustness was further examined through modality ablation analyses using the full multimodal feature set, a model without EMG features, and a model without both EMG and EDA features.

Given the large number of pairwise comparisons, *p*-values were corrected within each family of related analyses using the Benjamini–Hochberg false discovery rate (FDR) procedure. Separate corrections were applied to CORGIS dimensions, EARL emotion ratings, IDN/IEQ dimensions, physiological group comparisons, and single-feature regression analyses. Primary inferences were based on the FDR-corrected results, while the *p*-values reported in all the tables have been corrected.

## 3 Results

Based on the data collected from the two experimental conditions, the differences between the GamePlay and GameWatch groups were further analyzed. To provide a more complete understanding of the effect of interactivity in the emotionally challenging game scenario, both questionnaire data and physiological data were examined. The results are presented in two parts. The first part describes the subjective experiences reported by participants, and the second part presents the physiological responses during emotional-challenge-related events.

### 3.1 Results of questionnaire measures

#### 3.1.1 Perceived game challenge

Table 2 cnge experiences of participants in both groups. For perceived emotional challenge, the differences were marginally significant [*t*_(26)_ = 1.84, *p* = 0.08, 95% CI (–0.06, 1.03)], with interactivity enhancing the experience, being higher in Gameplay (*M* = 4.31, *SD* = 0.63) than in GameWatch (*M* = 3.83, *SD* = 0.75).

**Table 2:** Participants' perceived challenge experiences measured with CORGIS.

Dimension	Cronbach's α	GamePlay		GameWatch		*t*	*p*	95% CI	Cohen's *d*
		*M*	*SD*	*M*	*SD*				
Cognitive challenge	0.92	3.40	1.11	2.40	1.19	2.31	0.04*	(0.11, 1.90)	0.88
Emotional challenge	0.64	4.31	0.63	3.83	0.75	1.84	0.08	(–0.06, 1.03)	0.70
Physical challenge	0.85	3.91	0.80	2.81	1.04	3.13	< 0.01*	(0.38, 1.82)	1.18
Decision making	0.84	4.47	0.58	2.67	0.58	8.22	< 0.01*	(1.35, 2.25)	3.14

The differences in perceived cognitive challenge [*t*_(26)_ = 2.31, *p* < 0.05, 95% CI (0.11, 1.90)] and physical challenge [*t*_(26)_ = 3.13, *p* < 0.05, 95% CI (0.38, 1.82)] under the two conditions were significant. The intensities of perceived cognitive challenge (*M* = 3.40, *SD* = 1.11) and physical challenge (*M* = 3.91, *SD* = 0.80) in Gameplay were significantly more intense than those in GameWatch, with cognitive challenge (*M* = 2.40, *SD* = 1.19) and physical challenge (*M* = 2.81, *SD* = 1.04). As expected, the impact of interactivity on decision making was also significant [*t*_(26)_ = 8.22, *p* < 0.05, 95% CI (1.35, 2.25)], with the experience of decision making (*M* = 4.47, *SD* = 0.58) in Gameplay being significantly more than that in GameWatch (*M* = 2.67, *SD* = 0.58).

#### 3.1.2 Emotional responses

**Figure 3:**
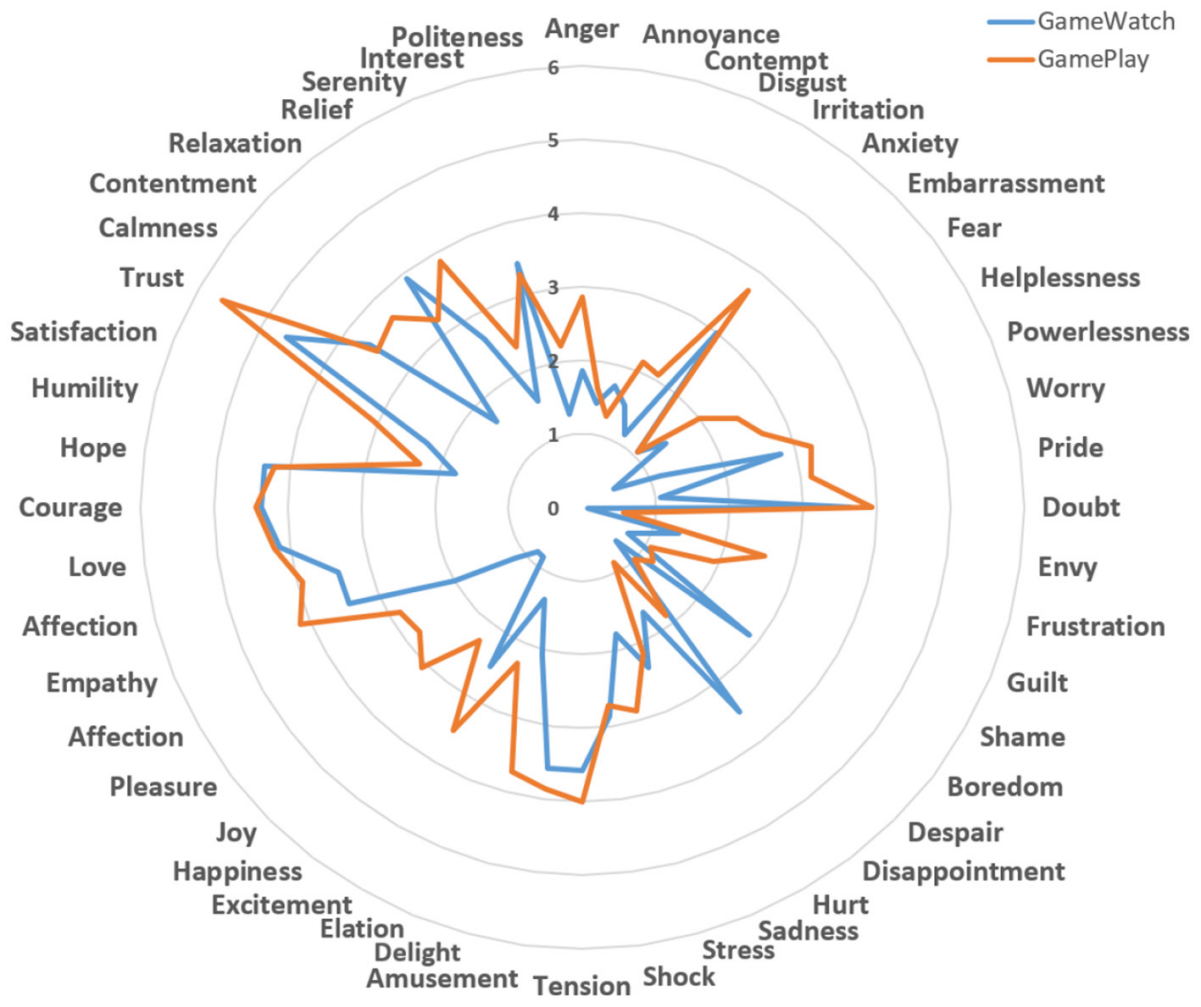
Participants' various emotional responses measured with the 48 EARL rating.

The induced emotional responses are presented in Figure 3. Results indicated that participants in both GamePlay and GameWatch groups reported experiencing multiple types of emotions. Using a rating score threshold of 4 (a score of 4 is generally considered moderate, and above 4 is considered to indicate a strong emotional response), we found distinct patterns between the two groups. GamePlay participants demonstrated strong emotional responses including trust, courage, love, hope, empathy, affection, and tension. In contrast, GameWatch participants showed strong responses primarily for trust, courage, love, and hope.

**Figure 4:**
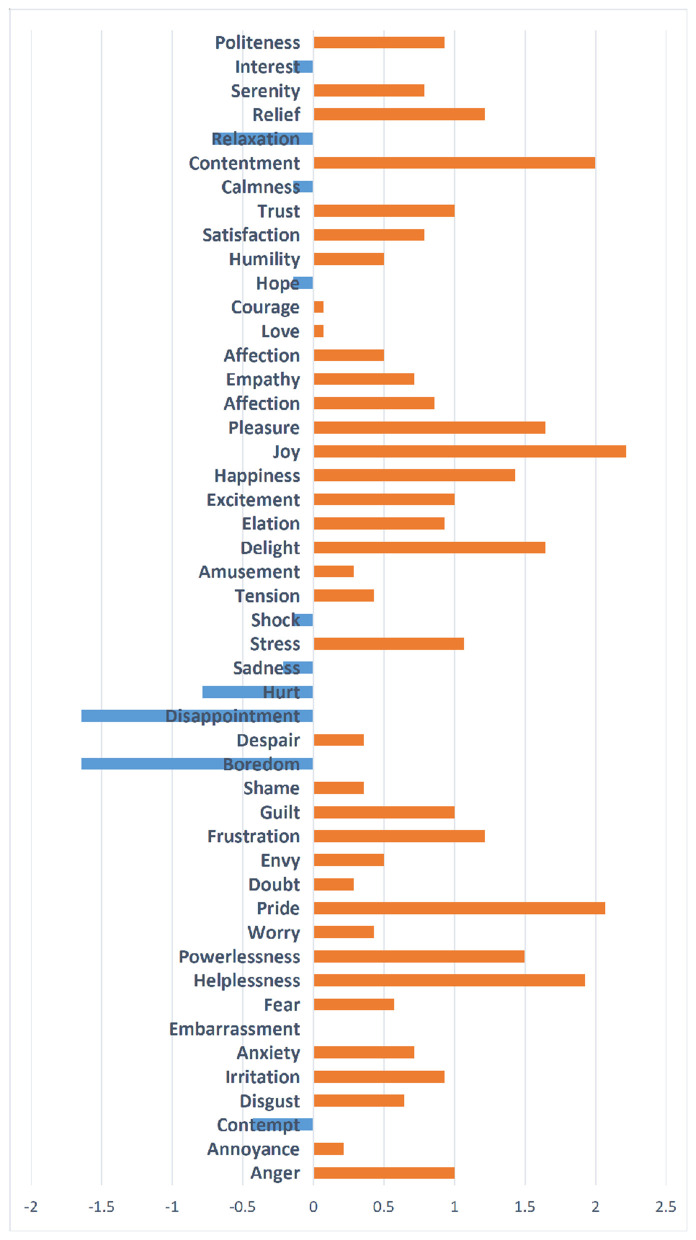
Rating score differences of emotional responses.

Specifically, to gain a deeper understanding of the differences between the GamePlay and GameWatch groups, we calculated the rating score differences for the 48 kinds of emotions. Figure 4 shows that, although no individual emotion remained statistically significant after FDR correction, the addition of interactivity in gameplay tended to amplify several emotional responses compared to game watching, particularly contentment, joy, pride, and helplessness. Conversely, interactive gameplay tended to reduce feelings of disappointment and boredom. For other positive emotional states, including pleasure, happiness, and delight, GamePlay participants reported moderately higher ratings than GameWatch participants.

#### 3.1.3 IDN-related user experiences and IEQ

In terms of aesthetic emotional experience, the reported data of the participants were in line with normal distribution and were analyzed using *t*-test. As shown in Table 3, after FDR correction, the Gameplay group showed significant differences in the aspects of role identification [*t*_(26)_ = 2.63, *p* < 0.05, 95% CI (0.26, 2.17)] compared to the GameWatch group. The difference in impression [*t*_(26)_ = 2.23, *p* = 0.08, 95% CI (0.08, 1.97)] and aesthetic pleasantness [*t*_(26)_ = 2.04, *p* = 0.08, 95% CI (0.00, 1.39)] approached marginal significance. There were no significant differences between the two groups with aspects of appreciation and suspense.

**Table 3:** IDN-related UX and IEQ.

Dimension/category	Cronbach's α	GamePlay		GameWatch		*t*	*p*	95% CI	Cohen's *d*
		*M*	*SD*	*M*	*SD*				
IDN-related UX									
Appreciation	0.80	4.12	0.77	3.93	0.97	0.58	0.57	(–0.49, 0.87)	0.22
Impression	0.87	3.88	1.14	2.86	1.29	2.26	0.08	(0.08, 1.97)	0.84
Suspense	0.37	2.90	0.83	2.62	0.99	0.83	0.53	(–0.43, 1.00)	0.31
Role Identification	0.77	4.19	0.98	2.98	1.42	2.63	0.04*	(0.26, 2.17)	0.99
Aesthetic Pleasantness	0.81	4.19	0.70	3.50	1.05	2.04	0.08	(0.00, 1.39)	0.77
IEQ									
Immersion		3.84	0.67	3.16	1.15	1.92	0.14	(–0.05, 1.42)	0.73
Cognitive Involvement	0.89	3.98	0.84	3.50	1.31	1.16	0.26	(–0.37, 1.34)	0.44
Real-world Dissociation	0.78	3.80	0.88	3.22	1.25	1.40	0.26	(–0.27, 1.41)	0.53
Control	0.66	3.49	0.72	2.96	1.46	1.22	0.26	(–0.37, 1.42)	0.46
Emotional Involvement	0.90	3.98	1.07	2.93	1.55	2.08	0.05	(0.01, 2.08)	0.79
Challenge	0.30	3.84	0.82	2.86	0.84	3.13	< 0.01*	(0.34, 1.63)	1.18

As shown in Table 3, the overall immersion score did not differ significantly between the two groups after FDR correction, although the GamePlay group (*M* = 3.94, *SD* = 0.67) reported a higher immersion score than the GameWatch group (*M* = 3.16, *SD* = 1.15), and a marginal trend was observed [*t*_(26)_ = 1.92, *p* = 0.14, 95% CI (–0.05, 1.42)]. Among the IEQ subdimensions, the GamePlay group showed a significantly higher rating on challenge [*t*_(26)_ = 3.13, *p* < 0.05, 95% CI (0.34, 1.63)]. The difference in emotional involvement was at the threshold of significance after FDR correction [*t*_(26)_ = 2.08, *p* = 0.05, 95% CI (0.01, 2.08)]. No significant differences were found between the two groups in cognitive involvement, real-world dissociation, or control.

### 3.2 Results of physiological analysis

#### 3.2.1 Modality-specific responses

Interactivity was associated with significant physiological differences across all five modalities. Table 4 summarizes a set of representative and clearly discriminative features.

**Table 4:** Physiological differences.

Modality	Feature	GamePlay *M* (*SD*)	GameWatch *M* (*SD*)	*p*	95% CI	Cohen's *d*
ECG	bpm	78.09 (6.82)	74.16 (11.06)	0.02*	(0.79, 7.08)	0.43
	R std	0.17 (0.07)	0.27 (0.20)	< 0.01*	(–0.16, –0.06)	–0.71
	RR mean	0.78 (0.08)	0.83 (0.13)	< 0.01*	(–0.09, –0.02)	–0.53
	pwr TP	16.84 (3.99)	24.61 (22.38)	0.02*	(–13.26, –2.27)	–0.48
RSP	val mean	1.12 (0.44)	1.29 (0.27)	0.12	(-0.30, -0.05)	–0.47
	Diffprd std	0.73 (0.39)	0.58 (0.38)	0.14	(0.02, 0.28)	0.39
EDA	HF time sam	118.58 (75.19)	70.29 (52.21)	< 0.01*	(26.05, 70.53)	0.75
	HF frq sam	4.67 (2.88)	2.99 (2.18)	< 0.01*	(0.81, 2.56)	0.66
	HF frq pwr	9.24 (13.21)	4.67 (7.30)	0.04*	(0.90, 8.24)	0.43
EMG	Total Eng	262.18 (109.03)	371.77 (202.46)	< 0.01*	(–165.25, –53.92)	–0.68
	5–6 level (7) Eng%	0.06 (0.03)	0.05 (0.03)	0.03*	(0.00, 0.03)	0.47
TEMP	mean	0.48 (0.74)	0.26 (0.75)	0.12	(–0.03, 0.48)	0.30
	Diff std	0.00 (0.00)	0.00 (0.00)	0.12	(–0.00, 0.00)	–0.33

##### 3.2.1.1 ECG

ECG features indicate stronger cardiac activation and reduced variability during interactive play. Heart rate was higher in the GamePlay group (*M* = 78.09, *SD* = 6.82) than in the GameWatch group (*M* = 74.16, *SD* = 11.06), indicating stronger cardiac activation (*p* < 0.05, Cohen's *d* = 0.43). The mean RR interval was lower in GamePlay (*M* = 0.78, *SD* = 0.08) than in GameWatch (*M* = 0.83, *SD* = 0.13), indicating a faster cardiac rhythm (*p* < 0.05, Cohen's *d* = −0.53). Beat-to-beat variability was lower in GamePlay (*M* = 0.17, *SD* = 0.07) than in GameWatch (*M* = 0.27, *SD* = 0.20), showing reduced variability under interactivity (*p* < 0.05, Cohen's *d* = −0.71). Total spectral power was also lower in GamePlay (*M* = 16.84, *SD* = 3.99) than in GameWatch (*M* = 24.61, *SD* = 22.38), consistent with reduced overall power (*p* < 0.05, Cohen's *d* = −0.48). Together, these results suggest that interactivity elevates physiological activation while constraining beat-to-beat variability, consistent with a more engaged and focused state.

##### 3.2.1.2 RSP

RSP features suggest a directional pattern in which interactivity may be associated with shallower and less stable breathing regulation, although these effects were no longer statistically significant after FDR correction. Mean respiratory depth was lower in GamePlay (*M* = 1.12, *SD* = 0.44) than in GameWatch (*M* = 1.29, *SD* = 0.27), indicating shallower breathing during play (*p* = 0.12, Cohen's *d* = −0.47). At the same time, breathing-cycle variability was higher in GamePlay (*M* = 0.73, *SD* = 0.39) than in GameWatch (*M* = 0.58, *SD* = 0.38), indicating less stable respiratory timing (*p* = 0.14, Cohen's *d* = 0.39). Although these differences did not remain significant after correction, the overall pattern suggests that active gameplay may promote a more vigilant and effortful breathing mode, marked by shallower breathing and greater fluctuation during decision-relevant moments.

##### 3.2.1.3 EDA

EDA features show stronger phasic arousal under interactivity. High-frequency event counts in the time domain were higher in GamePlay (*M* = 118.58, *SD* = 75.19) than in GameWatch (*M* = 70.29, *SD* = 52.21), indicating more frequent phasic events during play (*p* < 0.05, Cohen's *d* = 0.75). Frequency-domain indicators showed the same pattern. High-frequency event counts in the frequency domain were higher in GamePlay (*M* = 4.67, *SD* = 2.88) than in GameWatch (*M* = 2.99, *SD* = 2.18), indicating stronger frequency-domain reactivity (*p* < 0.05, Cohen's *d* = 0.66). High-frequency spectral power was also higher in GamePlay (*M* = 9.24, *SD* = 13.21) than in GameWatch (*M* = 4.67, *SD* = 7.30), consistent with elevated phasic power (*p* < 0.05, Cohen's *d* = 0.43). These results indicate more frequent and stronger short-lived arousal responses when players actively interact with the game, consistent with interactivity amplifying moment-to-moment emotional and attentional reactions.

##### 3.2.1.4 EMG

EMG results differentiate overall muscle activation from fine motor engagement. Total EMG energy was lower in GamePlay (*M* = 262.18, *SD* = 109.03) than in GameWatch (*M* = 371.77, *SD* = 202.46), indicating reduced overall muscle activation during play (*p* < 0.05, Cohen's *d* = −0.68). In contrast, the proportion of energy in finer bands was higher in GamePlay (*M* = 0.06, *SD* = 0.03) than in GameWatch (*M* = 0.05, *SD* = 0.03), indicating greater fine motor engagement (*p* < 0.05, Cohen's *d* = 0.47). This pattern suggests that interactive play is less dominated by gross movement, while selectively increasing fine motor activity consistent with continuous control inputs.

##### 3.2.1.5 TEMP

Temperature features showed weaker but directionally consistent differences, although these effects were not statistically significant after FDR correction. Mean temperature was higher in GamePlay (*M* = 0.48, *SD* = 0.74) than in GameWatch (*M* = 0.26, *SD* = 0.75), indicating a non-significant upward trend (*p* = 0.12, Cohen's *d* = 0.30). Temperature fluctuation was slightly lower in GamePlay (*M* = 0.0018, *SD* = 0.0011) than in GameWatch (*M* = 0.0021, *SD* = 0.0011), suggesting a non-significant trend toward reduced variability (*p* = 0.12, Cohen's *d* = −0.33). Although these temperature-related differences were less pronounced than those observed for ECG, EDA, and EMG, they may still provide complementary information about peripheral physiological regulation under interactivity.

#### 3.2.2 Physiological discriminability

Discriminability was evaluated using logistic regression with 10-fold cross-validation. To avoid optimistic estimates caused by within-subject dependence across train and test splits, this cross-validation was performed participant-wise (all windows from a given participant were kept within the same fold). With all 80 multimodal features, accuracy reached 82.69%, as shown in Table 5. This performance supports the objective separation between the Play and Watch conditions based solely on physiological signals.

**Table 5:** Regression results with ablation.

Feature set	Acc
Full multimodal set (80 features)	82.69%
w/o EMG (63 features)	81.12%
w/o EMG & EDA (51 features)	76.06%

The separation was not explained by a single marker. Single-feature logistic regressions were less accurate. The best single feature reached 64.41% accuracy.

As shown in Table 6, the strongest individual predictors were dominated by ECG spectral power features. Total ECG spectral power achieved 64.41% accuracy (*p* < 0.05, β = 0.09). Total ECG spectral density power achieved 63.96% accuracy (*p* < 0.05, β = 0.04). Very-low-frequency spectral density and very-low-frequency spectral power showed comparable performance. Their accuracies were 63.77% and 63.18%. Statistical significance was retained in both cases (*p* < 0.05). Beat-to-beat amplitude variability, quantified by the median absolute deviation of the ECG R-peak amplitude, also contributed with accuracy reached 62.78% (*p* < 0.05, β = 5.22).

**Table 6:** Top five single-feature regression results.

Modality	Feature	*p*	β	Acc
ECG	pwr TP	< 0.01*	0.09	64.41%
ECG	psd TP	< 0.01*	0.04	63.96%
ECG	psd VLF	< 0.01*	0.00	63.77%
ECG	pwr VLF	< 0.01*	0.10	63.18%
ECG	R mad	< 0.01*	5.22	62.78%

This pattern indicates that a subset of ECG-derived markers captures a stable portion of the Play–Watch difference. However, the ceiling of single-feature accuracy remained far below the full multimodal model. A single physiological marker was therefore insufficient for reliable discrimination.

Robustness was examined through ablation analyses that targeted potential motor artifacts. Accuracy remained high after all EMG features were removed, reaching 81.12%, as reported in Table 5. Only a small reduction from the full model was observed. The separation between Play and Watch was therefore not dependent on muscle activity alone.

To test whether discrimination could be sustained without electrodermal arousal signals, both EMG and EDA were removed. Accuracy decreased to 76.06%, as shown in Table 5. Discriminability was still preserved using ECG, RSP, and TEMP features. The larger drop after removing EDA indicates a meaningful contribution from phasic arousal. The remaining performance indicates that EDA was not sufficient by itself. Together, these results support a multimodal physiological signature of interactivity that remains robust to motor artifacts.

## 4 Findings and discussion

This study examined the role of interactivity in emotionally challenging games, with a focus on perceived emotional challenge, mixed-affect emotional experiences, and physiological responses. The findings provide empirical support for the importance of interactivity in shaping users' experiences in such contexts. Although interactivity may not be necessary for emotional challenge to be perceived, it appears to strengthen this experience and to enrich users' emotional responses. Differences were also observed in physiological responses between the GamePlay and GameWatch conditions, and multimodal physiological features further supported the discrimination between the two conditions. These findings are discussed in the following sections.

### 4.1 The role of interactivity in perceiving emotional challenge

To examine whether emotionally compelling narratives alone could elicit perceived emotional challenge, the results suggest that simply watching others play an emotionally challenging game could also evoke such an experience to some extent. However, this experience was further strengthened when interactivity was involved.

This finding is consistent with (Toh, 2023)'s study, which identifies different interactional patterns that shape emotional engagement. In the present study, interactivity required participants to actively engage with the narrative, make decisions, and deal with morally difficult situations. Compared with passive viewing, this process placed greater cognitive and emotional demands on players. As they were not only exposed to the narrative but also involved in its development, a stronger sense of personal engagement was likely to be produced. This was particularly evident when players had to make choices that could influence character outcomes.

A further explanation may lie in the tension between personal values and in-game decisions. As noted by Cheung and Huang (2011), spectators can also appraise the meaning of game events and respond emotionally to them. However, players are required to act within these situations rather than merely observe them. This makes the appraisal process more immediate and more personally involving. When morally ambiguous choices have to be made, players may experience a stronger sense of conflict and responsibility, which in turn intensifies perceived emotional challenge. By contrast, participants in the GameWatch condition were emotionally engaged with the narrative, but they were not required to make decisions or bear their consequences.

Overall, the findings suggest that emotionally challenging narratives can evoke perceived emotional challenge even without direct interaction, but interactivity plays an important role in intensifying this experience. It appears to deepen both emotional involvement and cognitive engagement, making the experience more personally challenging for players.

### 4.2 Influence of interactivity on mixed-affect emotional experiences

Regarding the influence of interactivity on mixed-affect emotional experiences, the findings suggest that interactivity increased both the complexity and the range of participants' emotional responses. Compared with those in the GameWatch condition, participants in the GamePlay group reported stronger and more contrasting emotions. Their experiences were characterized by a mixture of positive and negative feelings, whereas the emotional responses in the GameWatch group were relatively more uniform and tended to be calmer.

The EARL results further indicate that gameplay was associated with more ambivalent emotional experiences. Participants in the GamePlay condition reported emotional states that reflected both engagement and tension, such as excitement together with helplessness, or trust together with frustration. Such mixed-affect experiences are likely to arise when players are required to make difficult decisions and to face the possible consequences of their choices. In these situations, feelings of satisfaction, accomplishment, or attachment to the narrative may coexist with doubt, concern, or regret.

These findings suggest that interactivity does not simply intensify emotional experience. It also broadens the emotional range of gameplay by enabling multiple emotions to be experienced at the same time. This pattern is consistent with previous work (Klimmt et al., 2012), which shows that interactive systems can evoke diverse emotional responses. In the present study, such diversity appears to be an important part of how emotionally challenging gameplay becomes more immersive and personally involving.

### 4.3 Physiological responses under interactive conditions

Regarding physiological responses under the two conditions, the findings suggest that interactivity was associated with a distinct physiological state in the GamePlay group. Compared with GameWatch, interactive play was characterized by higher cardiac activation, reduced beat-to-beat variability, shallower and less stable breathing, and stronger phasic electrodermal responses. These patterns are consistent with a state of greater autonomic engagement during gameplay. At the same time, it should be acknowledged that the two conditions also differed in other aspects, such as motor activity, cognitive workload, and interaction pacing, which may have contributed to the observed physiological differences.

A possible explanation is that interactive gameplay required participants to remain continuously engaged, make decisions, and respond to morally complex situations. Under these demands, higher heart rate together with reduced variability may reflect increased tension and attentional engagement. Stronger phasic electrodermal responses further suggest more immediate arousal during interaction. The respiratory pattern, marked by reduced depth and greater variability, may indicate less stable breathing regulation during play. Temperature differences were weaker, but they provided complementary evidence of peripheral physiological change.

The EMG findings also suggest that the physiological differences cannot be explained by motor activity alone. Although interactive play involved active control, the observed pattern was not one of globally increased muscle energy. Instead, fine-band EMG energy proportion increased while overall muscle energy decreased, which is more consistent with sustained fine motor engagement than with gross movement. This interpretation is further supported by the feature ablation analysis, in which classification accuracy remained relatively high after EMG features were removed.

Overall, these findings suggest that interactivity was associated not only with stronger subjective emotional challenge, but also with a measurable shift in physiological response patterns. In addition, multimodal physiological features showed potential for distinguishing between the GamePlay and GameWatch conditions. This demonstrates the viability of using game-derived physiological signals as objective digital biomarkers to assess user states in ecologically valid interactive contexts. Such an approach aligns with foundational propositions that gameplay data can serve as non-intrusive biomarkers for computational mental health modeling and continuous well-being monitoring (Mandryk and Birk, 2019). Furthermore, our findings contribute to a broader research ecosystem that is increasingly leveraging multimodal physiological and affective data from real-world gameplay—such as recent large-scale esports datasets (Behnke et al., 2025)—to advance automated affect detection and personalized player-centric interventions.

### 4.4 Limits and future work

This study has several limitations. First, the sample size and age range were relatively small, and both experimental groups were heavily male-biased (12 males vs. two females). Because males and females can exhibit distinct differences in autonomic nervous system reactivity (e.g., EDA and HRV) and emotional expressivity, this imbalance may skew our findings toward a male-dominated demographic. Second, the highly skewed nature of participants' in-game moral decisions (12 sparing vs. two executing) prevented us from analyzing how different narrative outcomes specifically influence physiological responses. Third, inherent confounds between the conditions—such as higher motor activity, increased cognitive workload, and autonomous pacing in the GamePlay group—mean our identified physiological biomarkers likely capture a holistic mix of emotional challenge, cognitive effort, and physical engagement, rather than emotional variance alone. Finally, the focus on a specific game type (*Fallout 4*) and reliance on subjective self-reports may restrict generalizability.

Future research should expand the sample size, ensuring balanced representation across genders, ages, and cultures to establish more universally generalizable biomarkers, while allowing for statistical comparisons of divergent in-game moral choices. To strengthen internal validity, future studies should employ yoked designs or active control conditions to isolate emotional responses from motor and cognitive confounds. Additionally, combining peripheral physiological measurements with behavioral analysis and advanced sensing techniques will provide a more comprehensive understanding of emotional mechanisms. Future work will also integrate validated affective stimuli to explore the relationship between normative arousal-valence profiles and the unique mixed-affect experiences induced by emotional challenge.

## 5 Conclusion

This study provides empirical evidence that interactivity in digital games shapes perceived emotional challenge, mixed-affect experiences, and multimodal physiological responses. Emotional challenge could be elicited under both watching and playing in emotionally compelling narratives. Interactivity acted as an amplifier, increasing the intensity of perceived challenge and promoting more hedonic experiences. At the physiological level, systematic differences were observed between GamePlay and GameWatch across modalities. Objective discrimination between the two conditions was also supported by multimodal physiological features, suggesting measurable biomarkers linked to interactivity. These findings underscore the importance of considering emotional design in interactive technologies and have implications for the design of interactive systems, providing empirical support for advancing personalized mental health monitoring and intervention tools. By understanding how interactivity affects subjective experience and physiological state, designers can create technologies that are more engaging and more responsive. For instance, games can be tailored to provide appropriate levels of interactivity to elicit desired emotional responses and support balanced engagement.

## Data Availability

The data supporting the conclusions of this article will not be made publicly available, as it contains sensitive and private information that could compromise the privacy of the research participants, and the study's ethical approval does not permit data sharing.

## Footnotes

1 Detailed walkthrough of the quests can be found at https://fallout.fandom.com/wiki/Fallout_4_quests.

2
https://www.biopac.com/

## Appendix A

**Table A1:** The 80 extracted physiological features.

Modality	Domain	Feature type	Metrics/sub-features
**ECG**	Time (HRV)	HR	BPM (Bit Per Minute)
		R	Mean, Std, MAD
		Diff R	Mean, Std, MAD, MAV
		RR	Mean, Std, MAD
		Diff RR	Mean, Std, MAD, MAV
	Frequency (HRV)	RR power	VLF, LF, HF, TP, LF/HF, LF norm, HF norm
		RR PSD	VLF, LF, HF, TP, LF/HF, LF norm, HF norm
**TEM**	Amplitude		Mean, Std, MAD
	Diff Amplitude		Mean, Std, MAD, MAV
**RSP**	Time	Amplitude	Mean, Std, MAD
		Diff Amplitude	Mean, Std, MAD, MAV
		Period	Mean, Std, MAD, BPM (Breath Per Minute)
		Diff Period	Mean, Std, MAD, MAV
**EMG**	Wavelet decomposition		1-7 Band power, Total power, 1-7 Band power%, Entropy, RMS
**EDA**	Time		LF SAV, HF SAV, VHF SAV
	Frequency		LF SAV, LF power, LF power Std, HF SAV, HF power, HF power Std, VHF SAV, VHF power, VHF power Std

Std, standard deviation; MAD, mean absolute deviation; MAV, mean absolute value; TP, total power or PSD of {VLF,LF,HF}; LF or HF norm, $\frac{\text{LF or HF}}{\text{total power or PSD of \{LF,HF\}}}$; Band power%, $\frac{\text{Band power}}{\text{Total power}}$; Entropy, - ∑ Band power% log Band power%; RMS, root mean square; SAV, sum absolute value.
